# Adapting a digital monitoring system for self-management to geriatric COPD rehabilitation: A participatory mixed method study

**DOI:** 10.1177/20552076251343782

**Published:** 2025-06-09

**Authors:** Suzanne M Debeij, Eléonore F van Dam van Isselt, Marise J Kasteleyn, Heleen V Krabben, Petra Siemonsma, Wilco P Achterberg, Miriam L Haaksma

**Affiliations:** 1 Department of Public Health and Primary Care, 4501 Leiden University Medical Center, Leiden, The Netherlands; 2 University Network for the Care Sector South-Holland, 4501 Leiden University Medical Center, Leiden, The Netherlands; 3 LUMC Center of Medicine for Older People, 4501 Leiden University Medical Center, Leiden, The Netherlands; 4 National eHealth Living Lab, Leiden, The Netherlands; 5 Viduet Health, Utrecht, The Netherlands; 6 Department of Health Care, 125778 University of Applied Sciences Leiden, Leiden, The Netherlands

**Keywords:** Pulmonary rehabilitation, digital health technologies, post-acute care, user-centered design, blended care, agile, eHealth

## Abstract

**Introduction:**

Self-management for patients with chronic obstructive pulmonary disease (COPD) is essential in preventing relapse and rehospitalization. This study aimed to adapt a self-management-supporting digital monitoring system that was designed for COPD patients at home, to older patients with COPD and their healthcare professionals during inpatient geriatric rehabilitation. It evaluated feasibility, usability, and adherence based on qualitative and quantitative data collected across three iterations.

**Methods:**

This participatory mixed-methods study, conducted between October 2022 and June 2023, included interviews with patients and focus groups with healthcare professionals on the usability and feasibility of the system. Qualitative data were coded and analyzed using the framework method. Quantitative data from the monitoring system were descriptively analyzed.

**Results:**

Five healthcare professionals and 10 patients participated. Adjustments to the system and its use were made between iterations. Most patients used the system frequently. Insight into energy balance and health status was described as valuable features in a digital monitoring system. Other important aspects were easy initiation and use of the system and integration into the treatment program.

**Discussion:**

While the digital monitoring system and its use underwent various adaptations, further changes such as integration into the treatment program are needed for the system to be usable and feasible during geriatric rehabilitation. To transfer an eHealth intervention for home use to use during inpatient rehabilitation, substantial adaptations and time are required.

**Impact:**

This study highlights the importance of tailored digital health systems, offering guidance on implementing eHealth solutions in complex settings such as geriatric COPD rehabilitation.

## Introduction

Chronic obstructive pulmonary disease (COPD) has a global prevalence exceeding 10% and ranks as the fourth leading cause of death.^1,2^ Smoking is COPD's primary causal factor, and its prevalence increases with age.^2^ In the Netherlands, over 33,000 individuals with COPD are hospitalized per year due to an acute exacerbation.^3^ An exacerbation is, according to the GOLD guidelines, marked by a sudden worsening of respiratory symptoms that results in additional treatment.^4^ Many people who had an exacerbation are readmitted shortly after discharge, with one in 10 being readmitted to the hospital within a month, and over three in 10 within a year.^5^ Each exacerbation has deleterious health effects, such as reducing health status, quality of life, functional status, and increased mortality risk.^6^ Consequently, reducing the exacerbation rate, preventing readmissions, and sustaining recovery after an exacerbation are critical to improving outcomes for the population with COPD.

In the post-acute phase, rehabilitation can be offered to improve outcomes after an exacerbation.^7^ It focuses on restoring functional abilities or enhancing remaining capabilities for older adults with, possibly various, disabling impairments.^8^ This rehabilitation is short-term and is carried out in a therapeutic environment and has a multidisciplinary approach. The rehabilitation treatment program is personalized, consisting of several modules such as control of symptoms, physiotherapy, occupational therapy, and psychosocial interventions.^9^ One key objective in geriatric rehabilitation is the optimization of self-management. Enhanced self-management skills improve the recognition and management of an exacerbation, coping responses, and a person's energy management.^10^ Optimized self-management for patients with COPD can effectively improve health outcomes, support the transition home after rehabilitation, and contribute to sustainable recovery as well as prevent readmissions.^9,11–13^

eHealth can be used effectively to support self-management and should be considered in continued care to reduce admission rates.^14^ A study by van Buul et al. validated an eHealth program called SmartCOPD, aimed at optimizing self-management for community-dwelling people with COPD.^15^ This digital monitoring system supports patients and their (informal) caregivers through a symptom diary that alerts them to possible exacerbations and, by tracking and providing feedback on physical activity. Its implementation led to a significant decrease in the number of exacerbations and days admitted to the hospital.^15^ Both a symptom diary and feedback on behavior can play an important role in COPD care and enhancing rehabilitation with eHealth.^16–18^ Online COPD symptom diaries are frequently incorporated in self-management supporting eHealth tools,^17^ as symptom monitoring might help to indicate exacerbations and can impact function and quality of life throughout the day.^19,20^ However, evidence on these tools remains inconclusive regarding general effect on health behavior and their ability to detect exacerbations.^18,21–23^ A multidisciplinary approach and social support in the use of eHealth are important for self-management support after discharge from hospital.^24^

The adoption of eHealth in older individuals is generally poor and results from studies are inconsistent because eHealth applications often do not match the older individuals’ skills, needs, and wishes.^25,26^ Furthermore, eHealth should be adopted not only by older individuals, but also by healthcare professionals who may experience digital literacy issues, barriers to adapting their methods of delivering care and dread investing their time.^26,27^ Personalized support and the involvement of stakeholders, such as patients and professionals, in developing, implementing, and evaluating eHealth interventions can help overcome these barriers and increase usability and feasibility.^28,29^ Personalized support for eHealth and its long-term use can start during inpatient rehabilitation to improve patients’ skills and confidence for the transition home. However, knowledge regarding the use of eHealth in this complex setting is scarce.^30^ Therefore, this study aims to adapt a digital monitoring system, in co-creation with stakeholders, to the context of inpatient geriatric COPD rehabilitation. The adaptation is evaluated in multiple iterations by qualitative and quantitative measures of feasibility, usability, and adherence.

## Methods

### Study design and participants

This study had a mixed-methods participatory design. It incorporates an agile science approach and includes various iterations with interviews including a think-aloud task, and focus groups. The CeHRes roadmap was used to structure the process.^29^ The study was declared to be outside the scope of the Dutch Medical Research Involving Human Subjects Act (WMO) by a non-WMO review board of the Leiden University Medical Center (study nr 22-3044).

All participants were recruited from a post-acute, inpatient geriatric rehabilitation center located in a metropolitan area in the Netherlands (Laurens Intermezzo Zuid, Rotterdam). Healthcare professionals (HCPs) and patients were invited to participate. Inclusion criteria for patients were admission to the geriatric rehabilitation ward as result from an acute COPD exacerbation and having access to a smartphone or tablet. Patients were excluded if they had a life expectancy of less than three months or if they showed severe cognitive impairment. HCPs had to be stakeholder as defined in this study to be included. All participants provided written informed consent prior to their participation. Participants did not receive remuneration for participation.

### Digital monitoring system

SmartCOPD is a CE-marked Class 1 digital monitoring system that is designed to recognize signs of an exacerbation in community-dwelling people with COPD. The system was developed by Viduet Health BV (formerly Medicine Men, Utrecht, the Netherlands) with input from patients and stakeholders.^31^ The digital monitoring system was compatible on both IOS and Android devices and consisted of two components: a digital exacerbation action plan (DEAP) for recognizing and preventing exacerbations, and a smartwatch to monitor patient activity (SMA). The digital monitoring system required internet connection to function. HCPs could view data from both components through a web-based HCP dashboard. In this study, various aspects and processes of the digital monitoring system were adapted in advance for the geriatric rehabilitation setting. Patients in this study used the digital monitoring system for approximately three weeks during their rehabilitation.

The DEAP, accessible via a standalone mobile application, included three features for this study: a daily question (BASE), a PLUS question, and a Dutch version of the Clinical COPD questionnaire (CCQ). Each questionnaire could be answered when it was available in the mobile application.

**Figure 1.**
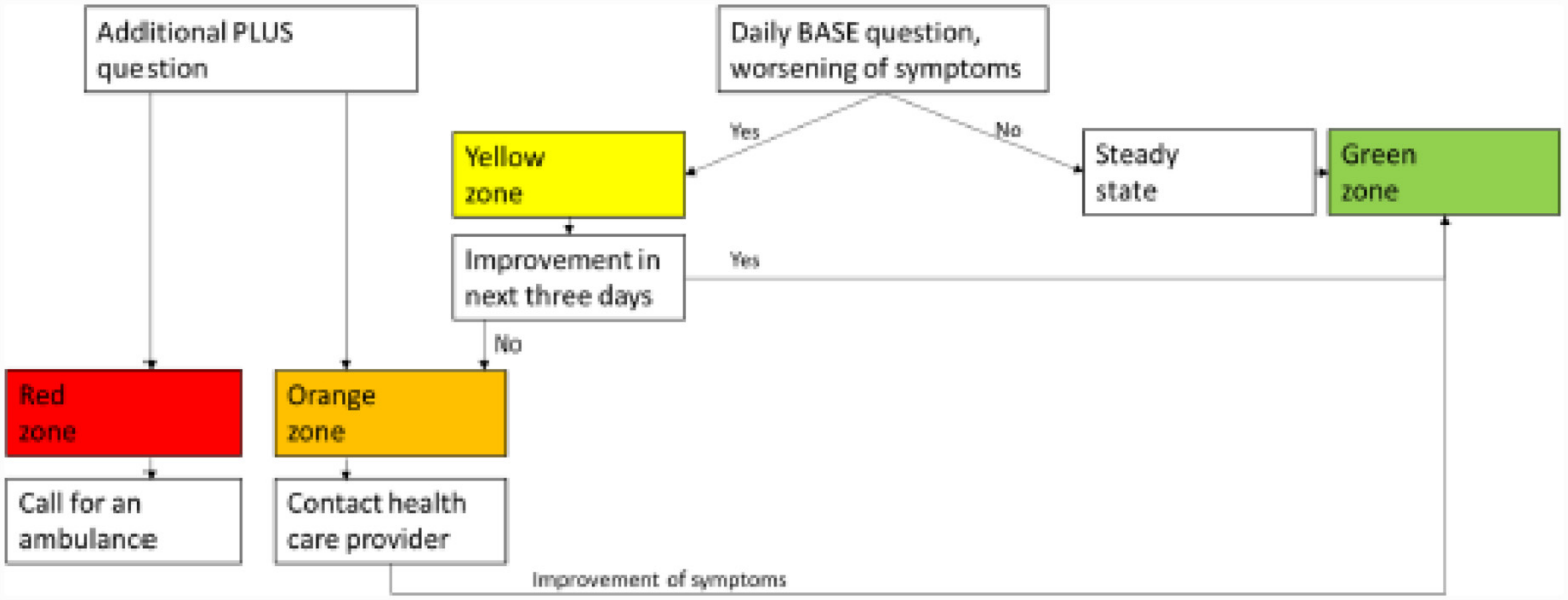
Visualization of the flow in zones of DEAP in the digital monitoring system.

Reproduced with permission from van Buul et al. (2021).^15^

- The BASE question was available daily. The question was “*I am experiencing an INCREASE in the following symptoms”* and was followed by checkboxes with eight symptoms of an exacerbation and an option of “*no worsening.”* Based on the answers, patients were assigned to one of four zones and received advice accordingly (Figure 1). In all zones except green, patients were advised to read their COPD action plan and react accordingly. When patients perceived a decrease in symptoms, they went back into the green zone. HCPs were notified if patients entered the non-green zones.
- The PLUS question had a similar set-up as the base question, was always available, and could be filled in if patients started to experience more severe symptoms. If they indicated a worsening of symptoms, they immediately entered the orange or red zone (Figure 1). The color of the zone was dependent on the symptoms a patient perceived.
- The CCQ was available multiple times a week. The CCQ is a ten-item questionnaire covering three domains of health: symptoms, functional state, and mental state, and has been proven useful and responsive in geriatric COPD rehabilitation evaluation.^32,33^ Answers to the CCQ are scored on a 7-point Likert scale. A higher score on the CCQ indicates lower health status (range 0–6). A score over two is interpreted as impaired health status.^33^

The SMA monitored the patient's daily steps, and patients could see their step count on their watch. It also contained a step goal set by HCPs for patients. Patients could also keep track of step counts during the day via a battery-shaped icon displaying their progress: green if the goal was reached, orange if the goal was close, and red if the step count was below target.

### Data collection

Qualitative data and quantitative data were collected in parallel.

#### Qualitative data

Ten persons conducted the qualitative data collection: two female researchers (SD, MH) and eight physiotherapy students (six female, two male). Researchers were experienced in interviewing, students were not. The patients had not met the interviewers before and did not know them; the HCPs had met the interviewers before. Participants were informed about the research objectives. No other people were present during the focus groups and interviews. To assess the digital monitoring system's usability and feasibility, the interviews and focus groups followed a predetermined semi-structured interview guide (Supplemental material 1) based on the CeHRes roadmap and the Optimized User Experience Honeycomb (Supplemental material 2)^.^^29,34,35^ The Honeycomb's seven facets shaped the questions to evaluate user experience.^34,35^ The interviews and focus groups were audio-recorded, and the recordings were stored in a secure and restricted data folder at Leiden University Medical Center. The interviewer made notes during the data collection.

The interview included a think-aloud task in which patients used the digital monitoring system and were asked to verbalize their thoughts while performing the task.^36,37^ The patients were asked about, for example, their experience with the digital monitoring system, the added value for their rehabilitation and points for improvement. An example of a question asked is “*How user-friendly do you find the application?*” The focus group included questions relating to the same topics as the interviews. In addition, the focus groups included statements to discuss such as “*I think it is feasible to use SmartCOPD in the treatment program of a patient in geriatric rehabilitation”.*

#### Quantitative data

The quantitative data consisted of baseline characteristics such as age, sex, GOLD stage and USER (Utrecht Scale for Evaluation of Rehabilitation) score,^38^ data from the questionnaires in DEAP (BASE, CCQ), data from the SMA (step count per part of the day and corresponding step goal) and answers to the system usability scale (SUS) questionnaire.^39^

Patients answered the SUS questions once after using the digital monitoring system. Responses were collected in an online platform by an HCP. The SUS is a reliable and robust instrument to measure the usability of new ICT systems or products.^39^ HCPs were invited to fill out the SUS in an online platform at each iteration. The 10-item questionnaire has a 5-point Likert scale ranging from “*strongly agree*” to “*strongly disagree*”. Total scores can range from 0 to 100, with higher scores indicating higher usability.

### CeHRes roadmap

**Figure 2.**
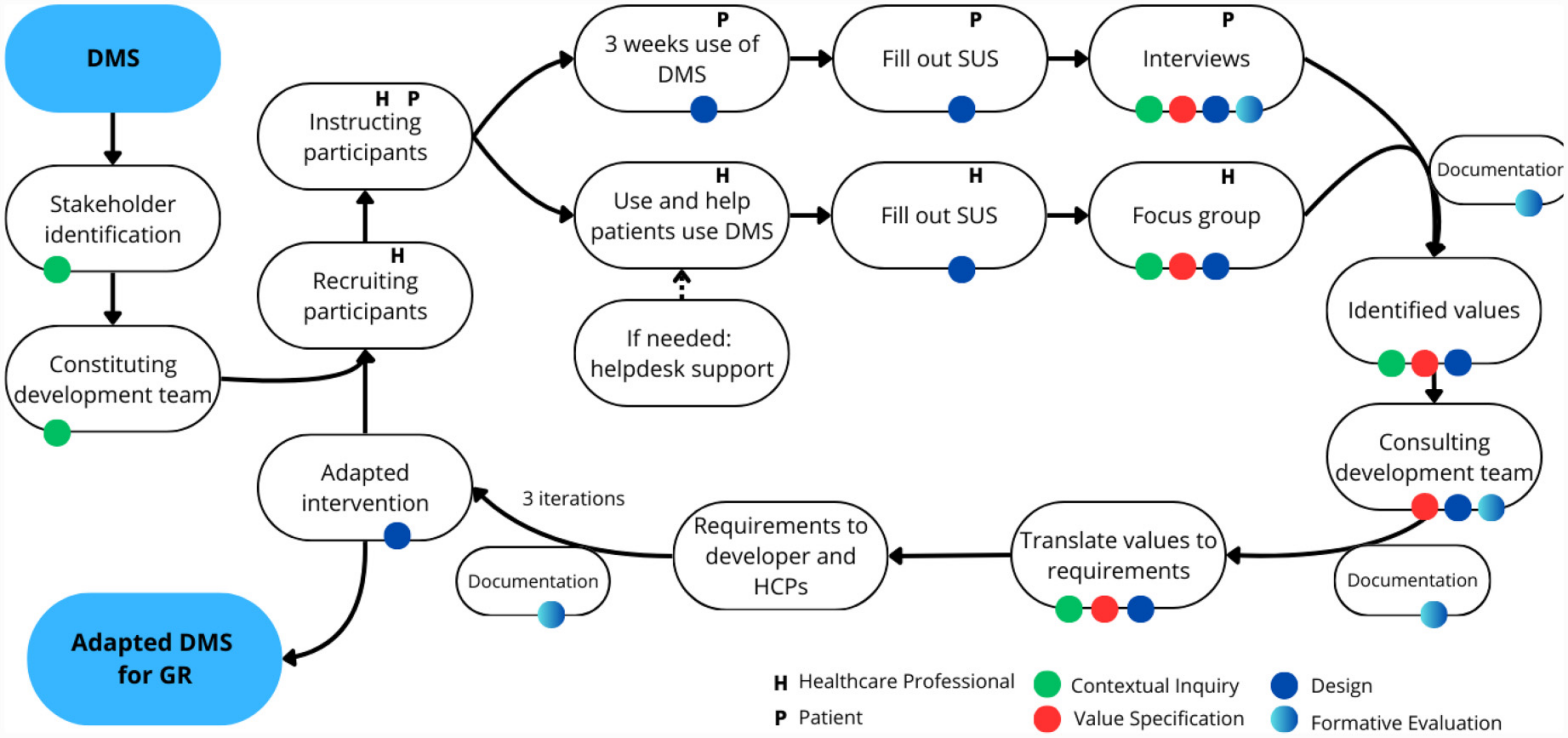
Visualization of the iterative phases of the study.

DMS: digital monitoring system; SUS: system usability scale; GR: geriatric rehabilitation; HCP: healthcare professional.

The CeHRes roadmap comprises five phases and continuous formative evaluation to guide the adaptation of the digital monitoring system. The first three of the five phases of the roadmap and the evaluation were applied in this study (see Figure 2). *Contextual Inquiry* is the first phase; the goal is gaining an understanding of the prospective users, other important stakeholders, the geriatric rehabilitation setting and the weak and strong points of the digital monitoring system. In the next phase *Value Specification*, the values of the key stakeholders are identified and prioritized based on their needs and wishes, and the added value of the digital monitoring system in geriatric rehabilitation is determined. Furthermore, the values are translated into requirements for the digital monitoring system. The third phase is the *Design* phase, in which the technology is adapted, and the usability is tested. See Table 1 for the content of the phases of the CeHRes roadmap within this study. The *Formative Evaluation*, in which the outcomes of the previous phases are checked, and the stakeholder perspective is included, was also covered.

**Table 1.** Research activities per phase of the CeHRes roadmap.

Phase	Goals	Set up in research	Deliverable
Contextual inquiry	Identify stakeholders Describe weak and strong points of current situation (usability and feasibility)	Identification of stakeholders ^40^ (only in iteration 1) Interview questions about the current situation Focus group questions about the current situation Development team provided future direction	Stakeholder overview Insight into weak and strong points of an iteration (usability and feasibility)
Value specification	Identify values and supposed added value of the digital monitoring system Prioritize values Translate values into technical requirements	Interview questions regarding needs and wishes (including Think-aloud task) Focus group questions regarding needs and wishes Development team translated values into requirements	Identified values of stakeholders (usability and feasibility) Prioritization of values and translation into requirements for change
Design	Develop and adapt digital monitoring system Test digital monitoring system Test persuasive elements	Requirements are implemented in the digital monitoring system Interview questions on usability based on the optimized user experience honeycomb Focus group questions based on the optimized user experience honeycomb	Insight into usability and feasibility New version of the digital monitoring system to be used in following iteration
Formative evaluation	Check whether outcomes of previous phases have been accounted for in the current phase. Continuously include stakeholder perspectives	Snowballing in stakeholder identification Documentation of interviews, focus groups and made changes Usability testing (Think-aloud task) Preliminary analysis per iteration Check outcomes of previous iteration Check if requirements address the values	Documentation and check-ups Preliminary analysis

### Procedure

Three iterations of data collection took place between October 2022 and June 2023. Figure 2 shows the content of the iterations. At the start of the study, stakeholders were identified based on the stakeholder identification process described by van Woezik et al.^.^^40^ After stakeholders were identified, a stakeholder development team was formed to manage the iterative phases of the development process. Next, HCPs were purposively sampled, approached, and informed about participation by a researcher. Participating HCPs then approached eligible patients and informed them of the study with a verbal explanation and an information letter. After signing the informed consent form, a patient participated for approximately three consecutive weeks during inpatient rehabilitation. The first week started with the installation of the digital monitoring system on smartwatch and smartphone. If the HCP or the patient encountered difficulties with the installation or the use of the digital monitoring system, they could reach out to a helpdesk by phone. After the first week, patients received a personalized step goal. For the next period, patients used the digital monitoring system. In the third week, patients completed the SUS once with an HCP. At the end of the three weeks, patients were interviewed. Interviews and focus groups were conducted at the rehabilitation center. HCPs were invited to fill out a SUS for each iteration. A focus group with HCPs was held at the end of each iteration.

After iterations one and two, a preliminary analysis was carried out to identify weak and strong points of the iteration, collect values, and map the usability and feasibility of the digital monitoring system for the participants. The development team prioritized these values and translated them into requirements. The requirements were passed on to a developer and the HCPs, who adjusted the digital monitoring system and its use in geriatric rehabilitation accordingly if this was possible in a short period of time. The adapted version for use in geriatric rehabilitation was used in the next iteration.

### Data analysis

#### Qualitative data

The interviews were coded and analyzed according to the principles of the Framework method.^41^ First, the audio recordings were transcribed, pseudonymized and not returned to the participant. One researcher (SD) and the students familiarized themselves with the data by checking and supplementing transcripts. After familiarization, a portion of the transcripts were thematically coded by one researcher (SD) in Atlas.ti 23. The same transcripts were also independently thematically coded in Word by another researcher (MZ) or the students. With these coded interviews, the codebook was developed by SD, who subsequently applied the codebook to the remaining transcripts. If necessary, new codes were added to the codebook. The data were then summarized and illustrated with quotations. Finally, the data were interpreted. The analysis was used to identify key elements and common themes in the interviews. Participants did not provide feedback on the findings. The process was supervised by two senior researchers (LvDvI and MK).

#### Quantitative data

Non-normally distributed data are described in terms of median and interquartile range. The step count per patient per day is visualized with a line graph. Adherence was operationalized as the percentage of days that steps were recorded (i.e., the smartwatch was worn), the percentage of days that the BASE question was completed in the period between first and last completed question and the percentage of days that the CCQ was filled out. All quantitative data were analyzed using SPSS version 29.

## Results

Five HCPs and 10 patients participated in the study. Four patients (patients 1–4) and four HCPs participated in the first iteration, two patients (patients 5 and 6) and four HCPs in the second iteration, and four patients (patients 7–10) and two HCPs in the third iteration. The median age [IQR] of the patients was 62 (20) years, eight of them (80%) were female, the median number [IQR] of days in rehabilitation was 38 (46) and their median USER score was 50 (IQR: 29). All patients were in GOLD stage 3 or 4. Patient characteristics are described in detail in Table 2. All patients reported being familiar with technology. They use it mainly in their spare time for contacting others and watching TV or news.

**Table 2.** Characteristics of participating patients.

Patient	Iteration	Sex	Age	GOLD stage	Days in rehabilitation	USER	Comorbidities	CCQ baseline
1	1	M	68	4	58	50	Heart failure	2.4
2	1	F	65	4	34	68		-
3	1	F	70	-	35	46	Malnutrition	1.9
4	1	M	51	4	21	70		2.3
5	2	F	61	4	37	41		1.1
6	2	F	71	3	34	45		1.4^a^
7	3	F	63	3	52	57		4.0
8	3	F	61	3	56	49		2.5
9	3	F	61	4	67	47	Heart failure, malnutrition	4.1
10	3	F	56	3	38	65		3.2^a^

-: Missing data; M: male; F: female; GOLD: Global Initiative for chronic Obstructive Lung Disease; USER: Utrecht Scale for Evaluation of clinical Rehabilitation; CCQ: Clinical COPD Questionnaire.

a CCQ at baseline is missing, first recorded CCQ is displayed.

Of the five (80%) participating HCPs, four were female. Two of the HCPs were physiotherapists, one was an occupational therapist, one was a certified nursing assistant, and one was a nurse practitioner. Their ages ranged from 32 to 55 years. They all reported being unfamiliar with the use of eHealth in the treatment of their patients.

Approximately 30 stakeholders were identified. Five of them formed the development team. The team consisted of an occupational therapist, a department head, someone from the purchasing department, an information manager, and a project leader from Viduet.

We describe the results for three time periods that the participants talked about: (1) at the initiation of the eHealth intervention, focusing on experiences during the installation process and related changes that were made between iterations; (2) use of the eHealth intervention during rehabilitation, with a focus on values, facilitators, and barriers to the eHealth intervention; (3) perspectives on the digital monitoring system after rehabilitation, including the possibility of using it at home. The changes made to the system or its use between iterations is described in Supplemental material 3.

### Initiation of the digital monitoring system

Most patients and the HCPs described the installation of the platform on their devices as a weak point of the digital monitoring system. This improved over the iterations. In the first iteration, all patients and all involved HCPs described difficulties with the installation process. The installation was mainly carried out by one HCP who felt that the complicated and very time-consuming process made the app less accessible, feasible, and usable. Because of the difficulties, the helpdesk was often needed during installation. An HCP in the first iteration stated:

The installation really takes a lot of time, and you frequently get an error message. The helpdesk is easy to reach…. You need an hour with patients to get it all done, because each application has to be linked. (physical therapist, iteration 1)

The development team saw that a change was required in the installation process. They decided to prioritize this requirement by outsourcing the main part of the installation from an HCP to the helpdesk in the second iteration. In addition, further adjustments to the installation process were required after the second iteration. Changes were made such as a technical adjustment to simplify the flow of installation and an adjustment in use to regular calls instead of video calls. After these adjustments, only half of the patients in the third iteration described the installation as a weak point. Patients in iterations two and three described experiencing inconveniences in contact with the helpdesk. Both HCPs also described that the inconveniences in contacting the helpdesk had increased compared to the first iterations. The HCPs felt that the rough start decreased patients’ motivation to continue using the app.

On the other hand, the HCPs were positive about the changes made between iterations because these saved them a lot of time. In addition, the other half of the patients in the third iteration experienced a smooth installation process.

It was very easy to install for, even for me, although I don't know anything about it. But I did it over the phone with the person who installs the platform. That went great, so I am smarter than I thought. It was very easy, really good. (patient 8)

### Use of the digital monitoring system during rehabilitation

Participants described the values, facilitators, and barriers to using the intervention during rehabilitation. The development team changed the values to requirements when necessary and possible. Participants saw the most value in gaining insight into the energy balance of patients and into patients’ health status.

#### Value: insight into energy balance

One value of the digital monitoring system for HCPs that we found was having insight into the energy balance of patients. However, HCPs believed that insight into patients’ energy balance should not rely solely on step counts, as step tracking may not capture some movements and other activities that also use energy. Therefore, HCPs felt the SMA did not capture the energy balance well. On the other hand, patients saw value in keeping track of steps and often monitored their step count on their smartwatch throughout the day.

In the first iteration, all patients described the SMA as usable and feasible, stating that they were more aware of their health and their physical activity during the day. In contrast, the step goal was described less positively. Several patients in iterations one and three mentioned that they did not have a step goal or that it did not suit them. In fact, quantitative data from the smartwatch showed that only five out of 10 participating patients had a personalized step goal. HCPs thought that this lack of personalized goals was partly due to a change in staff between the first and second iteration. In the third iteration, HCPs mentioned that the personalized step goal felt like a rough estimate. They did not add a personalized step goal because they did not feel its added value.

In the second and third iterations, patients described the added value of seeing their step count and progress in their step count during rehabilitation. However, half of the patients also described that the step count was inaccurately measured and therefore felt less reliable. This barrier was also described by HCPs in all iterations. HCPs further mentioned that a fixed step goal for a patient contradicts what is taught in rehabilitation, namely that not only steps, but all activities (e.g., a doctor visit or ADL) require energy and that patients need to balance their energy throughout the day.

HCPs also expressed concern that the digital monitoring system did not provide a clear clinical picture of a patient because steps were too one-dimensional.

Of course it doesn't show that someone is totally worn-out after washing and dressing, which is what we would really like to see. (nurse practitioner, iteration 2)

Both patients and HCPs also described other points for improvement in the SMA. These included adding oxygen saturation, measuring activities instead of steps, adding how someone feels during the day or adding the Borg scale^42^ to provide insight into the patient's perceived burden activities and thus their energy balance. Although the development team felt that adapting the SMA was a priority, they saw no opportunity to make changes in the SMA between the first and second iteration due to the limited time. Between the second and third iterations, the Borg Scale was added to the digital monitoring system to provide more insight into various activities of the patient. The “bad day” button was added to reduce the step goal accordingly when patients had a “bad day.” However, only one patient reported using it. HCPs in the third iteration still did not think the SMA was very useful during rehabilitation. If the digital monitoring system were to be developed further, they would like to see a connection between the Borg Scale and the step goal. In this case, the step goal decreases after a patient added a strenuous activity into the Borg Scale.

**Figure 3.**
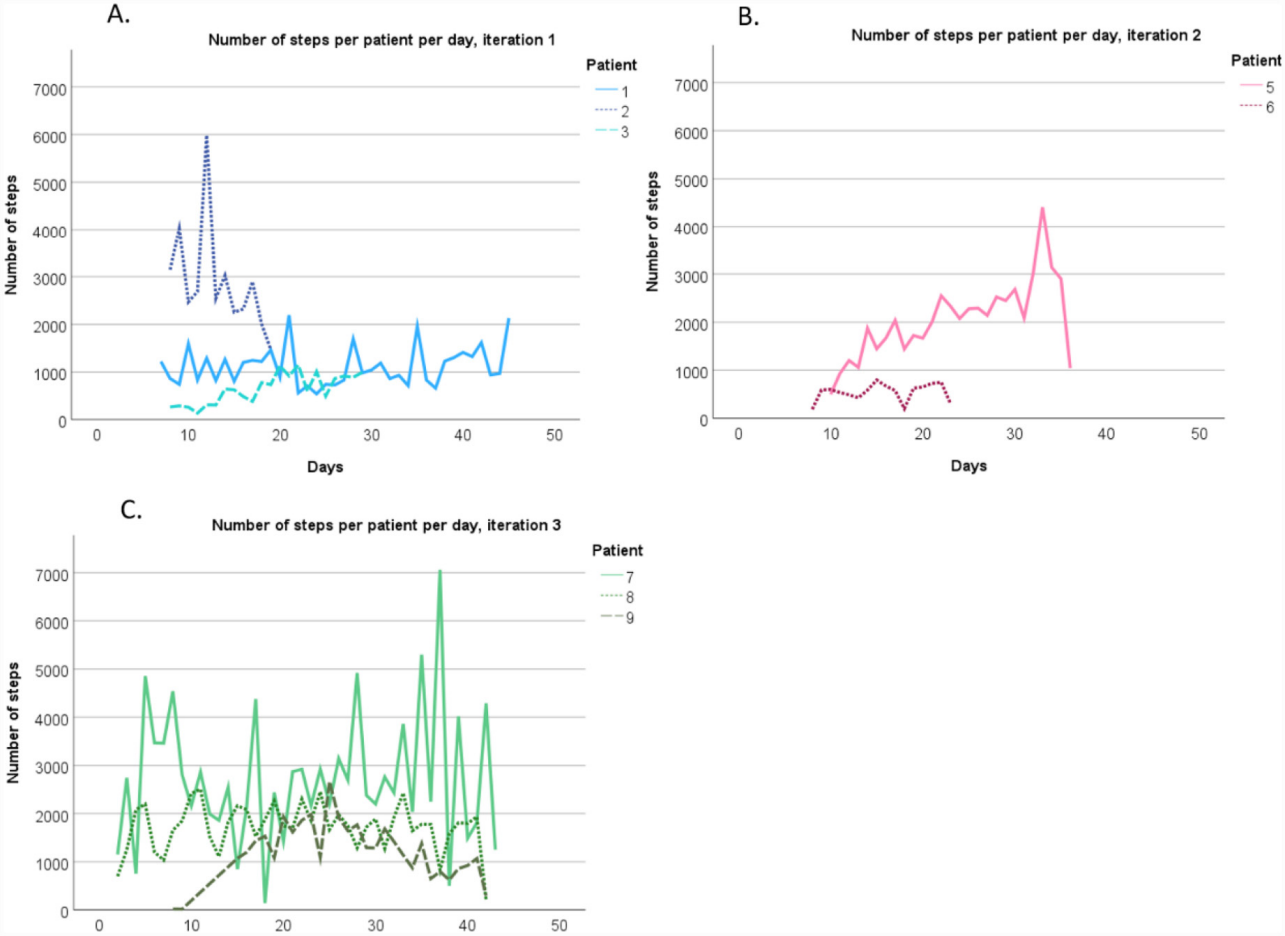
Number of steps during rehabilitation per person per day: (A) in the first iteration, (B) in the second iteration, (C) in the third iteration.

Quantitative data show that patients had a median step count of 1469 (IQR: 7041) during their rehabilitation (n = 8). The line graph shows that the measured step count varied per day and per patient (Figure 3). No clear trend could be derived from the data. Patients 1, 2, and 3 had a period of being isolated in their room due to COVID-19 infection during their rehabilitation. Patient 10 had no step count data due to a failed installation. All patients (n = 8) adhered to wearing their smartwatch between 62% and 98% of the days they participated.

#### Value: insight into health status

Another value that we found was the value of having insight into a patient's health status provided by DEAP. Barriers to using, and adherence to DEAP were also discussed.

In all iterations, HCPs felt that, within the digital monitoring system, DEAP had the most added value for patients in geriatric rehabilitation with COPD. HCPs described patients using SmartCOPD as being more aware of their health status.

I am really enthusiastic about the COPD action plan [DEAP]. You are really on top of it. Also, patients with that color. You give them insight, so you are actually digitizing the COPD action plan. I think that's fantastic. (physical therapist, iteration 3)

In addition, the HCPs indicated that they could use the patients’ responses to the DEAP BASE question to personalize patients’ therapy and that they themselves were aware more quickly of a patient's symptoms. However, several HCPs in the first iteration found the BASE question unclear and discussed improvement possibilities. The lack of clarity of the BASE question was also mentioned by some patients. They described ambiguity in questions in general, in the DEAP zones or in the corresponding alerts. The development team changed the value into a requirement and adapted the question after the first iteration. However, some lack of clarity remained.

Patients also described some other weak points in the DEAP. Some of them felt that the exacerbation risk alert was shown too quickly, or they wished that they could have their questionnaire back after completing it. Finally, a small number of patients did not think the app was desirable because the BASE question was the same every day.

In half of the interviews, patients reported that they answered the DEAP BASE question daily and almost all the patients found the DEAP easy to find and easy to use. They thought the DEAP was feasible. Some patients mentioned the convenience of the digital monitoring system being linked to their paper COPD action plan, or receiving an alert when they had symptoms.

I do like having it, because it warns you when something is wrong. I like that, it means you do not have to call a doctor right away. (patient 8)

The high adherence patient described in the BASE question was consistent with the quantified measurements of adherence. Four of the 10 patients had 100% adherence, three between 80 and 90%, one 53%, and two between 15 and 25%. For one of the last two, the installation was not completed. In addition to adherence, the CCQ showed a variance in adherence ranging from zero completed CCQs to 100% adherence.

##### Value: blended care

Another value that we found was the value of offering a digital monitoring system blended into the treatment program, instead of an addition to it.

HCPs discussed not only the content of the digital monitoring system during the focus groups, but also the way it was set up in the rehabilitation center. Both HCPs in the final iteration described that the digital monitoring system was not integrated into the treatment program but used alongside the treatment program. They felt the intervention would have been more useful if it had been adopted by the whole ward or even more widely, rather than being carried out of by a few HCPs, and if it had been integrated into the treatment program. The HCPs in all iterations also described other factors of usage that reduced the accessibility of the platform for HCPs as well as patients. This includes the mentioned inaccuracy of the step count, the change in actively involved HCPs during the research, difficulties in estimating and finding eligible patients, and the patients having other priorities. This reduction in accessibility also reduced feasibility.

Because now, it's sort of a separate thing, like ‘Oh, this is a patient, and he happens to have a question. Do we need to respond to that?’ Or it sorts of fades into the background, whereas if it's a fixed element in the treatment program, I think we could be more on top of it than we have been so far. (physical therapist, iteration 2)

Some patients experienced a lack of involvement of the HCPs. Slightly less than half of the patients explicitly stated that the use of the digital monitoring system was not discussed much with the HCPs.

##### General remarks

In addition to the values and barriers mentioned above, participants described some other values and formulated general remarks that influenced the usability and feasibility of the eHealth intervention.

Examples of other values that were mentioned by one or several patients were the added value of measuring heart rate (read from the smartwatch), the feeling of being in control, and the short lines of communication with the HCPs. Apart from these values, most patients indicated having doubts about the added value of the digital monitoring system for their rehabilitation. However, most patients would recommend the system to others even though they thought that it would be beneficial for a different target group (e.g., at the beginning, when you live alone, for younger people). Also, most patients briefly mentioned the design of the system. Most of them reported that the smartwatch was comfortable to wear, and more than half of the patients thought that the app providing DEAP was easy to use, and that the font was clear. In addition, half of the patients felt the DEAP app was user-friendly. However, some patients mentioned a lack of clarity in the general use of the digital monitoring system, which reduced usability. Furthermore, a small proportion felt that the app was not user-friendly or was boring.

So far everything is going really well, it's a positive experience. I just miss a small piece. Other than that, the questions are unambiguous and clear. I can fill it out neatly. And I try to be as, just as honest as possible. (patient 1)

So I fill it out, and the questions disappear, my answers are gone, and I can't find anything again. So, when I open the app now, it's the same as yesterday and the day before and the day before that. It's just a boring app. (patient 5)

HCPs did not describe any additional value beyond those described above. In their opinion, the added value of a digital monitoring system for patients should be mainly in three things: that they are aware of their symptoms, that their activities are visualized throughout the day, and that they learn about the changes to their body due to a recent exacerbation. They did not feel the digital monitoring system provided this. Furthermore, according to both HCPs in the third iteration, the difficulty finding eligible patients also resulted in low integration, making the system less feasible. They indicated that the low eligibility could be due to the age of patients, severity of their disease, social class, education level, and digital literacy in rehabilitation. They felt the system might be better suited for patients in a more stable environment. Therefore, they concluded that, for now, the implementation of the current version of the digital monitoring system in the geriatric rehabilitation setting was not feasible.

Also, HCPs in the first iteration mentioned the complexity of the digital monitoring system dashboard for HCPs. The HCP who mostly used the dashboard in the first iteration felt that the HCP dashboard was not intuitive and that it took a lot of clicks to get where you wanted to go. The development team considered what changes were required, and after these were made, the HCP in the final iteration thought the HCP dashboard was comprehensible and clear. However, the three HCPs who used the digital monitoring system most frequently still felt that the current version was not user-friendly enough to be implemented in geriatric rehabilitation. They thought it was too time-consuming for them and their patients.

Substantial variance in experienced usability was observed among patients, with SUS scores ranging from 7.5 (for the patient whose installation failed) to 97.5. All HCP scores were below 70, ranging from 42.5 to 67.5.

### Perspectives on the use of the digital monitoring system after rehabilitation

The possibility of using the digital monitoring system at home after rehabilitation was discussed both in the interviews and in focus groups.

Half of the patients mentioned that it would be useful to use the digital monitoring system at home after rehabilitation. They thought it was mostly useful to stay in contact with the HCPs at the rehabilitation center and to be alerted in case of an exacerbation.

HCPs in the first two iterations also saw value in using the digital monitoring system at home. They thought it would be valuable to teach patients how to use the digital monitoring system in the rehabilitation center so that they could use it at home. The HCPs mentioned that the most usable and feasible part for home use was the continued use of DEAP. In addition, they stated that it would be useful to know what kind of activities patients were doing at home, that the step goal should be adapted to the home setting and that a personalized step goal might be of more value in this setting. HCPs also mentioned that if they use the digital monitoring system at home, patients should be aware that they themselves are in control and must act when needed, not an HCP. HCPs are no longer responsible for patients after rehabilitation. They also discussed who should be responsible for the data provided by the digital monitoring system. They further described some logistical barriers to using the digital monitoring system at home, such as getting the smartwatch back and the period of time that the digital monitoring system should be used.

In the third iteration, both HCPs discussed whether the digital monitoring system would be more usable and feasible if it was implemented during a stable phase in a patient's COPD, instead of during rehabilitation. They thought that steps might be more beneficial at home, and that the app could be valuable in the stressful transition home if it was more integrated into a person's daily life.

A transition home from here sometimes is of course stressful for people, like ‘how am I going to do this at home’. They see bumps in the road. I can imagine that if people are in a more stable situation, so for example a long-term admission of just getting started from the home situation, where all goes relatively well, that that is less stressful for people. (physical therapist, iteration 3)

## Discussion

The aim of this study was to adapt a digital monitoring system to the needs and wishes of older individuals with COPD and their HCPs in inpatient geriatric rehabilitation. While the digital monitoring system and its use were adapted in several ways, further changes to both are needed to make the system usable and feasible in the geriatric rehabilitation setting. Professionals were most enthusiastic about the DEAP feature, while patients saw value in tracking step counts.

The HCPs viewed the DEAP as the most promising and valuable part of the digital monitoring system because of its ease of use and the insight it provides into a patient's health status. HCPs felt this was a usable and feasible feature. A narrative review emphasizes this added value, highlighting that patient experienced outcome measures significantly impact outcomes valuable for both patients and health care providers.^43^ Unfortunately, the study that showed a significant decrease in the number of exacerbations in patients who used the digital monitoring system compared to patients who did not,^15^ did not provide details on which aspects of the digital monitoring system contributed to this effect, nor did they elaborate on user perspectives. There is no consensus on the use of eHealth support for self-management by tracking health status during geriatric rehabilitation. Some studies state that eHealth programs that track symptoms in patients with COPD are both feasible and usable,^44–46^ while others are less convincing.^18,24^ Additionally, patients may have been less convinced because of the design of the system (as could be seen in low SUS scores), or because patients who believe that they are aware of their symptoms may not see any benefit to intensive monitoring.^47^ Perhaps the use of a self-management monitoring system is only feasible for a selection of patients with COPD during inpatient rehabilitation.

The finding that eHealth support for physical activity can have positive effects in patients with COPD^48^ is consistent with the value described by patients in the present study. The HCPs’ wish for a more multidimensional perspective, by expanding the step count with other factors such as heart rate, saturation or perceived exertion rate, is shared by others in various settings to gain more insight into energy costs.^49–52^ To our knowledge, such multidimensional accuracy has not yet been established in a wearable device, certainly not for people in geriatric rehabilitation. However, energy management techniques may be of particular importance for people with low energy levels.

The technical issues experienced by participants can be divided into two parts. First, they described reduced confidence in the step count due to inaccurate measurement, making it less usable. This is in line with other literature.^45,53^ The inaccuracy of the measurement could be increased due to sensor placement, possible gait abnormalities, slow walking speed, functional limitations, and different body morphologies.^54,55^ Second, due to difficulties in the installation process, the system was not easy to use initially, which reduced its feasibility. Both technical and credibility issues have been described as major barriers to the use of eHealth interventions for COPD management.^56^ However, technical installations at the beginning might be described as a smaller barrier when patients use a device for a longer period.

Although blended self-management interventions may potentially improve health outcomes in patients with COPD,^14^ the digital monitoring system was not optimally integrated into the inpatient geriatric rehabilitation program. This may be due to the small number of HCPs involved and their lack of experience with eHealth. eHealth programs that successfully combine eHealth and non-eHealth treatment can complement each other and are more beneficial in geriatric rehabilitation than programs that use it as addition to the treatment.^30,48^ This preference is also described by prospective users of eHealth in geriatric rehabilitation^57^ and patients with COPD.^58^ Additionally, the integration of the information from the digital monitoring system in patients’ medical records could increase accessibility to the data,^26^ which might also increase ease of use, and can increase quality of care.^59^ If a digital monitoring system is more integrated into the treatment with more health status indicators, is easier to use from the start and measures accurately, the system may be a better fit for inpatient geriatric rehabilitation.

### Strengths and limitations

Following the CeHRes roadmap, this study conducted multiple iterations of qualitative and quantitative data collection. The multiple iterations and different research methods provided more in-depth insight into the participants’ perspectives and allowed for reflection on the changes that were made over time. In addition, the use of the User Experience Honeycomb model made it clear to the development team which values should be prioritized for change between iterations. This research also had some limitations that should be mentioned. Because this was a single-center study, the results may be less generalizable to other settings or countries. Also, the short time between iterations influenced the adjustments that could be made, resulting in fewer adjustments than desired. Moreover, it was difficult to find eligible patients, which resulted in a small sample size and thus less robust evidence. The small sample size could be due to reasons such as “empty beds” in the rehabilitation center or a target group that was too vulnerable to participate, which may be attributed to geriatric challenges. Participants who chose to take part might have had a greater interest in eHealth, potentially introducing selection bias. However, the critical feedback provided by the patients suggests this was not the case. Besides, no medical practitioner was included as HCP which might show a lack of practitioner perspective. However, the role of the medical practitioner in this setting was minimal because the nurse practitioner acts in the role of medical practitioner in this setting. Finally, there were continuity issues due to COVID-19 and changes in the HCP team which might influence the use of the digital monitoring system for some participants.

## Conclusion

This study highlights the aspects that should be considered when transferring a digital monitoring system for COPD self-management to a complex setting like inpatient geriatric COPD rehabilitation. Professionals were most enthusiastic about the DEAP feature, whereas patients saw value in tracking their step count. After several iterations with adaptations to the system and its use, further changes are needed for it to be usable and feasible in this specific setting. For successful adoption, the system must be easy to use from the start, it must measure accurately, and there must be sufficient support and knowledge in the entire team. In addition, the digital monitoring system should be integrated into the rehabilitation process to complement it. The differences in perspectives of different stakeholders underline the importance of involving all stakeholders. In future research, the DEAP and SMA could be further adapted to inpatient geriatric rehabilitation to be usable and feasible. Additionally, a larger sample across various geriatric rehabilitation centers, a longer study duration, and an expansion of objective measures, such as assessments of symptom improvement, are recommended. It should be recognized that potentially major adaptations and therefore multiple iterations are needed to adapt an eHealth intervention to another setting, especially to a difficult context as geriatric rehabilitation. Finally, stakeholders’ perspectives on implementing not just an eHealth program, but a multidimensional blended-care program to support self-management during inpatient geriatric rehabilitation should be explored.

## Supplemental Material

sj-docx-1-dhj-10.1177_20552076251343782 - Supplemental material for Adapting a digital monitoring system for self-management to geriatric COPD rehabilitation: A participatory mixed method study Supplemental material, sj-docx-1-dhj-10.1177_20552076251343782 for Adapting a digital monitoring system for self-management to geriatric COPD rehabilitation: A participatory mixed method study by Suzanne M Debeij, Eléonore F van Dam van Isselt, Marise J Kasteleyn, Heleen V Krabben, Petra Siemonsma, Wilco P Achterberg and Miriam L Haaksma in DIGITAL HEALTH

sj-docx-2-dhj-10.1177_20552076251343782 - Supplemental material for Adapting a digital monitoring system for self-management to geriatric COPD rehabilitation: A participatory mixed method study Supplemental material, sj-docx-2-dhj-10.1177_20552076251343782 for Adapting a digital monitoring system for self-management to geriatric COPD rehabilitation: A participatory mixed method study by Suzanne M Debeij, Eléonore F van Dam van Isselt, Marise J Kasteleyn, Heleen V Krabben, Petra Siemonsma, Wilco P Achterberg and Miriam L Haaksma in DIGITAL HEALTH

sj-docx-3-dhj-10.1177_20552076251343782 - Supplemental material for Adapting a digital monitoring system for self-management to geriatric COPD rehabilitation: A participatory mixed method study Supplemental material, sj-docx-3-dhj-10.1177_20552076251343782 for Adapting a digital monitoring system for self-management to geriatric COPD rehabilitation: A participatory mixed method study by Suzanne M Debeij, Eléonore F van Dam van Isselt, Marise J Kasteleyn, Heleen V Krabben, Petra Siemonsma, Wilco P Achterberg and Miriam L Haaksma in DIGITAL HEALTH

sj-docx-4-dhj-10.1177_20552076251343782 - Supplemental material for Adapting a digital monitoring system for self-management to geriatric COPD rehabilitation: A participatory mixed method study Supplemental material, sj-docx-4-dhj-10.1177_20552076251343782 for Adapting a digital monitoring system for self-management to geriatric COPD rehabilitation: A participatory mixed method study by Suzanne M Debeij, Eléonore F van Dam van Isselt, Marise J Kasteleyn, Heleen V Krabben, Petra Siemonsma, Wilco P Achterberg and Miriam L Haaksma in DIGITAL HEALTH
