# Supplementary material for: Adapting a digital monitoring system for self-management to geriatric COPD rehabilitation: A participatory mixed method study
Source: Digit Health. 2025 Jun 9;11:20552076251343782. doi: 10.1177/20552076251343782 (PMC12174668; doi:10.1177/20552076251343782)
Supplement: sj-docx-1-dhj-10.1177_20552076251343782 - Supplemental material for Adapting a digital monitoring system for self-management to geriatric COPD rehabilitation: A participatory mixed method study [file sj-docx-1-dhj-10.1177_20552076251343782.docx]

# Supplemental material 1. Translated interview guide and focus group guide.

## 1a. Interview guide

**Introduction**

"Good morning, thank you for being available for the interview today. My name is .... For the past weeks, you have been using the *digital monitoring system* on your smartphone and smartwatch. We are curious about your experience. In this interview, we will explore how easy it is to use the app. We will start with a part called 'think aloud'. We will ask you to open the app and answer your daily question. You will tell me what you are thinking as you perform the action in the app. We will practice this beforehand with a practice task. After the 'think aloud', you can put the phone down and we will ask you questions about how easy it is to use the app. The entire interview will take between half an hour and 45 minutes and will be recorded for further analysis. In the analysis, your data will be anonymized, and the material will not be shared outside the research team, you will be completely anonymous in the analysis. You may withdraw from the study at any time. Are you okay with the interview being recorded?

I'll start the recorder in a moment and ask you this question again, so it's also on the recorder.

*-start recording-*

Are you okay with the interview being recorded?"

**General questions**

- How long have you had COPD?
- How did you end up on this rehabilitation unit?
- What do you do normally do with your smartphone or tablet?

**Use of SmartCOPD**

Thank you for your answers. The next part is 'think aloud'. The idea is that you use *the digital monitoring system* and share your thoughts out loud while using it. You are allowed to share both the pleasant and unpleasant user experiences. We will first practice this with an exercise. The practice task is as follows: look up on your mobile phone what the weather will be like today. While you're doing this, you can say out loud what you are thinking.

*-execution of the exercise assignment-*

Thank you very much. We are now going to do the same type of assignment, but with you answering the daily question in the *digital monitoring system*.

*-execution think aloud-*

**Key questions**

That was the think aloud. We will now move on to the interview questions. These questions are about the use of SmartCOPD, the application, the smartwatch, and your experiences with the platform. When you're ready, we'll start with the first question.

THEME A: Experiences

- What are your experiences with using the *digital monitoring system*?
- What obstacles did you encounter while using *the digital monitoring system*?
- What is your experience with searching for something in the app?
- What are your needs when using an app like this?
- What are your preferences when using an app like this?
- What is the added value of the app for you during your rehabilitation?
- Do you trust the information the app provides? Why or why not?

THEME B: SmartCOPD

*Application:*

- What do you think of the style/design of the app?
  - What do you think of the font?
  - What do you think of the font size?
  - What do you think of the colors?
  - What do you think of the sound?
  - What do you think of the notifications?
- How user-friendly do you find the app?

*Smartwatch:*

- What do you think of the style/design of the smartwatch?
  - What do you think of the color?
  - What do you think of the wearing comfort?
  - What do you think of the design?
  - What do you think of the font?
  - What do you think of the font size?
  - What do you think of the number of notifications in a day?

*Future:*

How can the ease of use of the app be improved?

What would you like (to be different) in the app?

Are there any other issues you encounter when using the app?

What would be the added value of *the digital monitoring system* if you could use it at home?

Why should you or shouldn't you recommend *the digital monitoring system*?

**Final questions**

These were the interview questions. To sum up briefly, for you the advantages are: *-summarize advantages-*, the disadvantages of the use of *the digital monitoring system* are*: -summarize disadvantages-*. You would like to see the following changes in the future *-summarize-.*

- What are the main points we discussed today for you?
- Is there anything else that you feel has not been discussed and that could be important for our research?
- May we approach you in the future – if necessary – for follow-up research?
- Is there anything else you'd like to share?

If you think of anything else later, you can pass it on to the research team or your healthcare professionals. We want to thank you very much for your participation in the study. You will soon be able to return your smartwatch to …. Thanks again.

## 1b. Focus group guide

**Introduction**

"Good [morning/afternoon], thank you for being available for today's focus group. My name is ... And these are my colleagues [name] and [name]. For the past weeks, the patients have been using the *digital monitoring system* on their smartphone and smartwatch, and you have been guiding them. I'm curious about your experience. In the focus group, we want to discuss the usability and feasibility of the app in the geriatric rehabilitation setting. We will do this based on statements or questions. The focus group will last an hour to an hour and a half and will be recorded for further analysis. In the analysis, your data will be anonymized, and the material will not be shared outside the research team, you will be completely anonymous in the analysis. You may withdraw from the study at any time.

I will start the recording in a moment and ask you all if you are okay with the conversation being recorded. Are there any people who don't like it?"

-start recording-

"Are you okay with the conversation being recorded?"

**General questions [*only in first focus group*]**

- I would like to ask you all to introduce yourselves. Can you please explain your role within the organization?
- What is your experience with eHealth in the treatment program of a patient in geriatric rehabilitation?

**Key questions**

We would like to get a clear picture of your experiences with *the digital monitoring system*.

*General/Content:*

- What has been your experience with *the digital monitoring system* in the past weeks?
- What is your need for using a platform like this in the treatment program?
- What is your preference in using such a platform in the treatment program?
- Statement: It is feasible for me to use t*he digital monitoring system* in the treatment program of a patient in geriatric rehabilitation.
- Statement: I find it useful to use *the digital monitoring system* in the treatment program of a patient in geriatric rehabilitation.
- How user-friendly do you find *the digital monitoring system*?
- What do you think are barriers to using *the digital monitoring system*?
- What do you think are facilitators when using t*he digital monitoring system*?
- Why would you, or would you not recommend *the digital monitoring system* to other healthcare professionals and rehabilitation centers?

*Detail:*

- Do you trust the information provided by *the digital monitoring system*?

I would like to get a clear picture of what you think is important for the future of *the digital monitoring system*.

*Future:*

- What would you like to keep of *the digital monitoring system*?
- What would you like to change about *the digital monitoring system*?
- What could be done differently in the platform to improve user-friendliness?
- How can we apply/improve the use of *the digital monitoring system* in this rehabilitation center?
- What could be done differently in *the digital monitoring system* to better align with the care goals for the patient in terms of self-management?
- What would motivate you to continue using this platform?

**Final questions**

We are now coming to the end of this focus group. We will first briefly summarize the main points of the focus group and then ask you the final questions.

*-Summary-*

- What are the main points we discussed today for you?
- Is there anything else that you feel has not been discussed and that could be important for our research?
- Are you open to follow-up research?
- Is there anything else you'd like to share?

The results of the focus group will be used for the further development of the application. So, some adaptations will be made for the next iteration. If you think of something you would like to share with us later, you can contact us by email *-mail address-* or telephone *-number-.* We would like to thank you very much for your participation in this focus group and look forward to seeing you again in the next round.
