# Supplementary material for: Adapting a digital monitoring system for self-management to geriatric COPD rehabilitation: A participatory mixed method study
Source: Digit Health. 2025 Jun 9;11:20552076251343782. doi: 10.1177/20552076251343782 (PMC12174668; doi:10.1177/20552076251343782)
Supplement: sj-docx-2-dhj-10.1177_20552076251343782 - Supplemental material for Adapting a digital monitoring system for self-management to geriatric COPD rehabilitation: A participatory mixed method study [file sj-docx-2-dhj-10.1177_20552076251343782.docx]

# Supplemental material 2. The optimized User Experience Honeycomb model.

The User Experience Honeycomb Model by Morville contains seven facets to describe user experience: useful, usable, desirable, findable, accessible, credible, valuable^1^. The optimized version by Karagianni divided the seven facets in three categories: use, feel and think^2^.

For the digital monitoring system (DMS) in this study, the seven categories can be explained as follows:

- Useful: *Is the DMS beneficial, does it fulfill a need?*
- Valuable: *Does the DMS help people get closer to their goal?*
- Credible: *Can the DMS be trusted and believed?*
- Desirable: *Does the DMS fit with needs and interests?*
- Findable: *Is the information in the DMS easy to find?*
- Usable: *Is the DMS easy to use?*
- Accessible: *Is the DMS manageable for individuals in geriatric rehabilitation?*

**References**

1. Morville P. User experience design, <http://semanticstudios.com/user_experience_design/> (2004).

2. Karagianni K. Optimizing the UX honeycomb; a small amendment to the classic diagram hopefully improves its UX, <https://uxdesign.cc/optimizing-the-ux-honeycomb-1d10cfb38097> (2018).
