# Supplementary material for: Adapting a digital monitoring system for self-management to geriatric COPD rehabilitation: A participatory mixed method study
Source: Digit Health. 2025 Jun 9;11:20552076251343782. doi: 10.1177/20552076251343782 (PMC12174668; doi:10.1177/20552076251343782)
Supplement: sj-docx-3-dhj-10.1177_20552076251343782 - Supplemental material for Adapting a digital monitoring system for self-management to geriatric COPD rehabilitation: A participatory mixed method study [file sj-docx-3-dhj-10.1177_20552076251343782.docx]

# Supplemental material 3. Adaptations made in (use of) the digital monitoring system

| **Date** | **Old situation** | | **New situation** | |
| --- | --- | --- | --- | --- |
| 19-12-2022 | R1 | Registration is done by an HCP | R2 | Registration is initiated by the HCP and carried out by the patient and the helpdesk.  Patients receive a letter informing them what they need to bring with them to the online appointment with the helpdesk (smartwatch, mobile phone, access to mail, access to Wi-Fi, a self-made password). They call the support agent for installation. If it does not work out, the HCP will take over. |
| 19-12-2022 | R1 | DEAP base question: I am experiencing an INCREASE in the following symptoms | R2 | DEAP base question: Compared to my NORMAL situation, I experience an INCREASE in the following symptoms.  When the HCP explains the app, he/she explains to the patient that the question is a comparison with normal, not with the situation yesterday |
| 19-12-2022 | R1 | Dashboard HCP: The HCP had to get to know the system to familiarize him/herself with the system. A navigation bar with several slide-out menus was used. | R2 | New HCP dashboard: Where the HCP can immediately see what they are there for (e.g., steps count, DEAP status). There are buttons and clickable fields instead of a navigation bar. |
| 19-12-2022 | R1 | No view of the DEAP status on the HCP dashboard. | R2 | An extra tile on the HCP dashboard containing the DEAP status and average number of steps. |
| 07-01-2023 | R2 | New dashboard HCP without DEAP status and button | R2 | New dashboard HCP with button to go directly to DEAP |
| 01-01-2023 | R1 | HCP had to check if the postal code of patient was correct. | R2 | Automatic postal code check during patient registration |
| 01-01-2023 | R1 | Date of birth had to be entered as mm/dd/yyyy | R2 | Automatic date of birth fix. It is now possible to click on the date via the calendar. |
| 12-02-2023 | R1 &R2 | The start screen of the app is a screen for blood pressure testing (not applicable for this study) | R2/3 | The start screen of the app is DEAP. |
| 17-01-2023 | R2 | Registration is done by the patient. Patients receive a letter from the HCP telling them what they need to have ready. They then **video** call the Medicine Men support agent for installation. If it doesn’t work out, the HCP will take over | R3 | Registration is done by the patient. Patients receive a letter from the HCP telling them what they need to have ready. They then call the Viduet support agent (by phone) for installation. If it doesn't work out, the HCP will take over |
| 12-2-2023 | R2 | Installation Fitbit and Viduet app according to the steps in round 2 | R3 | Installation Fitbit and Viduet app according to the new flow developed by Viduet.   - 1 device - easier for the participant - flow consisting of 4 steps - automatic notification if something has gone wrong and what is going on, with both the patient and Viduet Support. |
| 12-2-2023 | R2 | Manual for installation without the new flow | R3 | Revamped guide on how to install the smartwatch from the Viduet app. |
| 12-2-2023 | R1/R2 | The only insight into the energy a patient used is gained through the number steps someone takes. | R3 | The Borg scale and the 'I'm having a bad day' button have been added to *the digital monitoring system* to obtain a more multidimensional view. |
| 03-04-2023 | R2 | Patients only use Viduet in the rehabilitation center | R3 | Rehabilitators use Viduet in the rehabilitation center and at home.  Before they go home, patients are called by Viduet to adjust the data to the home situation. This is also the moment when where it is a buddy can be added. |
