# Supplementary material for: Adapting a digital monitoring system for self-management to geriatric COPD rehabilitation: A participatory mixed method study
Source: Digit Health. 2025 Jun 9;11:20552076251343782. doi: 10.1177/20552076251343782 (PMC12174668; doi:10.1177/20552076251343782)
Supplement: sj-docx-4-dhj-10.1177_20552076251343782 - Supplemental material for Adapting a digital monitoring system for self-management to geriatric COPD rehabilitation: A participatory mixed method study [file sj-docx-4-dhj-10.1177_20552076251343782.docx]

**Mixed Methods Reporting in Rehabilitation & Health Sciences (MMR-RHS)**

**Instructions:** The following checklist outlines essential information for mixed methods reporting. **1)** **Indicate “Y” if the standard is *fully met* or “N” and additional comments if lacking**. **2) Document page number where element is located**.

| **Title** | **Y/N;**  **Comments** |
| --- | --- |
| Concisely describes the topic of the study identifying the study as mixed methods | Y; 1 |
| **Abstract** | **Y/N;**  **Comments** |
| Summarizes key elements using *journal specific* abstract format; For example: Introduction, Methods, Results, Discussion, and Significance/potential impact to rehabilitation and/or societal health | Y; 1 |
|  | **Y/N; Page #**  **Comments** |
| **Introduction** |  |
| Includes literature review on the topic of interest (quantitative, qualitative, and mixed) | Y; 1, 2 |
| *Identifies gap that justifies the need for mixed methods approach* | Y; 2 |
| Clearly states overarching goal of the study that supports a mixed methods approach | Y; 2 |
| *States the rationale for using mixed methods research* | Y; 2 |
| Clearly identifies discrete aim(s) for qualitative and quantitative components  Aims align with corresponding component methods | Y; 2 |
| Provides statement of significance and potential impact | Y; 2 |

| **Mixed Methods Reporting in Rehabilitation & Health Sciences (MMR-RHS)** | |
| --- | --- |
| **Methods** |  |
| **Design –** *Clearly describes the mixed methods design* (exploratory sequential, explanatory sequential, concurrent, etc.) used to accomplish the overarching goal of the project:   - *Emphasis noted* (i.e., Sequential QUAL--> quan or QUAN--> qual; Concurrent QUAL + QUAN) - *Visual display of overall design highlighting integration* (e.g., model, flow chart, figure) | Y; 2, 5-7 |
| Describes and supports the qualitative and quantitative methodologies (phenomenology, randomized control trial) used to accomplish the discrete aim(s) of the project | Y; 3-7 |
| States researcher(s) background and contributions to project (e.g. content or methods expertise, relationships to participants) | Y; 4 |
| Identifies setting (e.g. hospital system, geographical location) | Y; 2,3 |
| **Subjects/Participants** - Clearly describes and supports the following:   - Sampling and recruitment - Inclusion/Exclusion criteria - Ethical considerations (consent process, researcher relationship with participants) | Y; 2, 3 |
| **Data collection** - Clearly describes and supports the following:   - Pilot study (if applicable) - Instrumentation (validity, reliability) - Implementation matrix (e.g. data source, timeline, type, anticipated outcomes) | Y; 4-7 |
| **Data analysis** - Clearly states and describes analysis procedures for:   - Qualitative - Quantitative - *Mixed Methods (integration)* | Y; 7 |
| **Methodological Rigor** – Clearly describes steps taken to establish rigor:   - Qualitative (e.g. credibility, dependability, confirmability, transferability) - Quantitative (e.g. validity, reliability, generalizability) - *Mixed Methods* (validity or legitimacy) | Y; 7 |
| **Results/Findings** |  |
| Clearly presents findings for study components:   - Qualitative (includes data exemplars) - Quantitative - *Mixed Methods-Provides integrated findings/overall study results* (e.g., joint display) | Y; 7-14 |
| **Discussion** |  |
| - *Incorporates discussion on implications of integrated findings* | Y; 14 |
| - Provides synthesis and interpretation of findings in the context of existing literature and theoretical/conceptual framework | Y; 14, 15 |
| - Includes subsection of limitations | Y; 15, 16 |
